# Effect of the Organic Production and the Harvesting Method on the Chemical Quality and the Volatile Compounds of Virgin Olive Oil over the Harvesting Season

**DOI:** 10.3390/foods9121766

**Published:** 2020-11-28

**Authors:** Ana I. Carrapiso, Aránzazu Rubio, Jacinto Sánchez-Casas, Lourdes Martín, Manuel Martínez-Cañas, Concha de Miguel

**Affiliations:** 1 Escuela de Ingenierías Agrarias, Universidad de Extremadura, Av. Adolfo Suárez s/n, 06007 Badajoz, Spain; arubiosa@alumnos.unex.es (A.R.); martinlu@unex.es (L.M.); conchademiguelgordillo@gmail.com (C.d.M.); 2 Centro de Investigaciones Científicas y Tecnológicas de Extremadura (CICYTEX), Instituto Tecnológico Agroalimentario de Extremadura (INTAEX), Av. Adolfo Suárez s/n, 06007 Badajoz, Spain; jacintojesus.sanchez@juntaex.es (J.S.-C.); manuel.martinez@juntaex.es (M.M.-C.)

**Keywords:** virgin olive oil, organic production, harvesting method, harvesting time, volatile compounds

## Abstract

Organic production has increasing importance in the food industry. However, its effect on the olive oil characteristics remains unclear. The purpose of this study was to research into the effect of organic production without irrigation, the traditional harvesting methods (tree vs. ground picked fruits), and the harvesting time (over a six-week period) on the oil characteristics. Free acidity, peroxide value, *K*_232_, *K*_270_, Δ*K*, total phenols, oxidative stability and the volatile compound profile (by SPME extraction, gas chromatography and mass detection) of olive oils from the Verdial de Badajoz cultivar were analysed. The organic production affected the peroxide value, total phenols, oxidative stability and 34 out of 145 volatile compounds. Its effect was much less strong than that of the harvesting method, which affected severely all the chemical and physical-chemical parameters and 105 out of 145 volatile compounds. Conversely, the harvesting time was revealed as a factor with little repercussion, on the chemical and physical-chemical parameters (only peroxide value was influenced), although it affected 83 out of 145 volatile compounds. The larger content in total phenols in the organic oils than in the conventional ones could explain the increase in oil stability and the differences in the volatile compounds.

## 1. Introduction

Virgin olive oil (VOO) is a valuable product obtained mechanically without any refining processes, so it keeps olive fruit compounds such as antioxidants [1] as well as compounds responsible for its typical colour and flavour. Olive oil flavour depends on the content in bitter-tasting compounds, such as phenolic compounds, but also on the volatile compounds, which are responsible for the typical odour notes and potential defects.

The most important VOO volatile compounds are formed through the lipoxygenase (LOX) pathway [2], C5 and C6 LOX compounds being the major contributors to the essential green sensory attribute [3]. When olives are released from the trees (either by falling down spontaneously or by harvesting them), progressive cell disruption takes place, which triggers the LOX pathway [3] and, therefore, the generation of C5 and C6 compounds and the development of the typical olive oil flavour. Factors affecting the activity of the enzymes involved in the LOX pathway, such as the fruit cultivar and the agronomic and processing conditions, may influence the volatile compound profile [2,3], and thus the flavour traits [4]. In addition, fruit decay favours chemical oxidation and microbial enzyme activity, which cause an increase in fermentation compounds, volatile phenols and chemical oxidation compounds [4], which are involved in most of the defective flavours of olive oil [3,5]. 

Over the last decades, organic agriculture has increased in importance. Regulation 834/2007 [6] (soon to be replaced) defines it as a system combining the best environmental practices, a high biodiversity level, the preservation of natural resources, animal welfare, and production methods based on natural substances and processes. Each State within the European Union have set up a control system to ensure that the organic agriculture comply with this regulation. It has been reported not to improve the sensory quality of food [7]. In the case of olive oil, results are not consistent. At small scale, it has been shown that restrictions in the use of chemicals (e.g., fertilisers) result in changes in the phenol content, some volatile compounds and sensory traits [8]. However, at large scale results do not seem to indicate a clear trend, which might be due to further differences in the agronomic practices [9]. In addition, little information about the effect of the organic practices on oil from unirrigated orchards and its volatile compounds is available [9], even though unirrigated and traditionally farmed systems tend to be based on more sustainable practices. In traditionally farmed orchards, harvesting is usually performed manually from the trees. Although not advisable for the production of high quality oil [10], harvesting the fruits from the ground is still resorted to in traditional systems when other methods are not feasible. It is currently performed when hand-picking is not convenient (then, the fruits are knocked down with a pole, e.g., when the fruits are scarce, scattered or difficult to access), or when the fruits fall down due to over-ripening or after climatic events such as strong winds. As ground fruits yield poor quality oils that usually cannot be marketed as virgin oil and need chemical refining, they are managed separately, and often undergo longer storage times, which favour further decay. It would be convenient to know if differences in the quality and the volatile compounds of oil when comparing both harvesting methods are consistent over the harvesting season. The harvesting time is of great interest for the industry. Early harvesting facilitates high prices in the market. However, as fruit ripeness increases, oil content rises, although in contrast oil quality worsens [11,12]. Little information is available about the effect of harvesting time on the volatile compounds, especially in the case of organic production [13].

About 1,800,000 tons of olive oil were produced in Spain in the 2018–2019 session [14]. The more abundant cultivars in Spain are Picual (over a million ha) and Hojiblanca (about 270,000 ha). Verdial de Badajoz is also among the main Spanish olive cultivars, with about 30,000 ha [15], being farmed mainly in the west (Extremadura region).

To date, most research about the effect of organic production and harvesting conditions has been focused on oil from irrigated orchards. More information on how these factors affect oil characteristics throughout the harvesting season is advisable to ensure the highest oil quality when there is no irrigation, which is more environmentally sustainable. Therefore, the aim of this study was to research the effect of organic production under traditional agronomic practices, the traditional harvesting methods (tree-picked vs. ground-picked fruits), and the harvesting time (over a six-week period) on the chemical and physical-chemical parameters and on the volatile compound profile of the olive oils produced from olives from the Verdial de Badajoz cultivar in a commercial olive mill. 

## 2. Materials and Methods 

### 2.1. Experimental Design and Samples

Olive fruits (*Olea europaea* L., Verdial de Badajoz cultivar) were collected from unirrigated trees grown in either organic or conventional orchards in the Lácara region (Badajoz, Spain). In the conventional production system, the fertiliser was a nitrogen-phosphorus-potassium-boron complex (20-8-14-0.1 B), as it is common practice in the region. In the organic production, the fertiliser was made up of weeds cut in springtime added to composted olive mill and pruning waste and hay. When mature (maturity index in the orchard ranging between 1 (fruits with green yellowish skin) and 5 (fruits with black skin and <50% purple flesh)), the fruits from the organic orchards were collected from the trees (Organic), whereas the ones from the conventional orchards were collected either from the trees (Conventional) or from the ground (Ground). They were mechanically processed, separately (in different days after proper cleaning to avoid cross-contamination), into oil under the same conditions in a local factory over the harvesting session. The organic production was subjected to the official control established in Spain according to Regulation 834/2007. Oil samples were taken from a tank filled during a week (20.000 L) for the Organic and Conventional oils, or directly from the production line for the Ground oil (which was produced once per week) once a week from the beginning of November to mid-January. Then, the eighteen (three types of oil x six weeks) virgin olive oils were kept at 6 °C and analysed.

### 2.2. Chemical and Physical-Chemical Analyses of Oil

The so-called quality parameters (free acidity, peroxide value, *K*_232_, *K*_270_, and Δ*K* extinction coefficients), which are taken into account to establish the olive oil categories within the European Union according to the Commission Regulation 2568/91 [16] and subsequent amendments, were determined [16]. 

The total polar phenol content was determined using the Folin-Ciocalteau colorimetric method [17]. The results were expressed as caffeic acid equivalent in mg·kg^−1^ oil.

The oxidative stability index (induction time expressed in hours) was determined by using an eight-channel 743 Rancimat instrument (Metrohm, Herisau, Switzerland), heating the oil samples (2.5 g) at 100 °C under an air flow of 10 L h^−1^ [18].

### 2.3. Volatile Compound Analysis

The virgin olive oil samples (5 g) were introduced into glass screw top vials, with laminated Teflon-rubber disks in the caps. The vials were left in a water bath at 40 °C for 10 min to equilibrate the volatile compounds in the headspace. Then, a solid-phase microextraction (SPME) needle was inserted through the disk, and a 1-cm 50/30 µm thickness DVB/Carboxen/PDMS fibre (Supelco, Bellefonte, PA) was exposed to the headspace for 40 min while the vial was kept in the 40 °C-water bath. Later, the fibre was transferred to the gas-chromatograph inlet (splitless mode, 250 °C). 

The chromatographic separation of the compounds was carried out using a HB-5 (50 m × 0.32 mm i.d, 1.05 μm) column (Agilent, Avondale, AZ, USA) placed into a gas-chromatograph (Agilent 6890 series) equipped with a mass spectrum detector (Agilent 5973). The oven temperature was held at 40 °C for 10 min and risen at 3 °C min^−1^ to a temperature of 160 °C, and then at 15 °C min^−1^ to a final temperature of 220 °C, where it was held for 10 min (total run time: 64 min). Mass spectra were generated by electronic impact at 70 eV, with a multiplier voltage of 1756 V. Data were collected at a rate of 1 scan s^−1^ over the 30–300 *m/z* range. The transfer line to the mass spectrometer was maintained at 280 °C. The Agilent MSD Chemstation software was used. *n*-alkanes (C5-C18) were analysed under the same conditions to calculate the linear retention indices (LRI). 

The identification was performed by matching mass spectra (MS) and LRI with those of reference compounds analysed under the same conditions (a total of 62 Sigma-Aldrich reference compounds were used), or with those included in the Flavornet (www.flavornet.org) or NIST [19] databases. Two samples of each oil batch were analysed, and results were expressed as total area counts.

### 2.4. Data Analyses

A three-way (organic production, harvesting method, and harvesting time) Analysis of Variance (ANOVA) was performed on the data. When a significant effect was found, the Tukey test was carried out to compare the means. A Principal Component Analysis was performed on the mean values for each sample to evaluate the relations among variables and samples [20]. The statistical analyses were performed by means of the SPSS version 22.0. 

## 3. Results and Discussion

### 3.1. Chemical and Physical-Chemical Parameters

Table 1 shows the results from the three-way ANOVA performed on the data from the chemical and physical-chemical analyses carried out on the Verdial de Badajoz virgin olive oils. The effect of the organic production was moderate (three out of seven parameters were affected), the harvesting method greatly influenced the parameters (all of them were affected), and the effect of the harvesting time was weak (only one out of seven parameters were affected) (Table 1).

#### 3.1.1. Effect of the Organic Production (Organic vs. Conventional)

**Figure 1:**
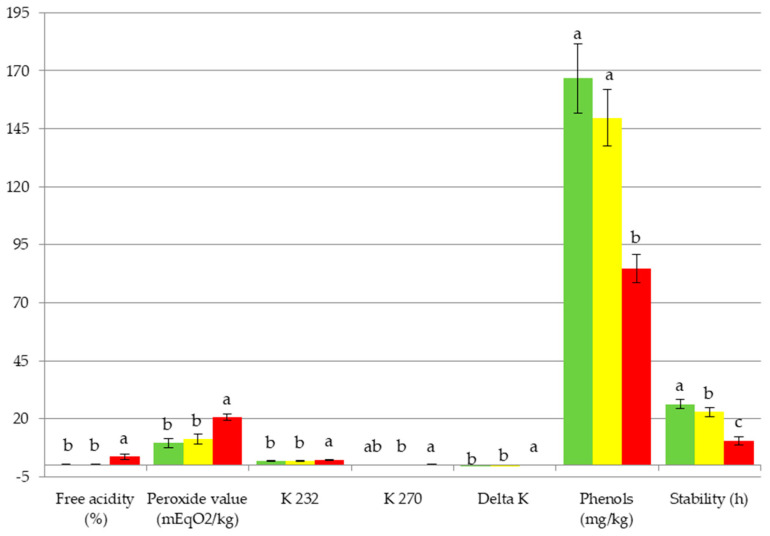
Mean values and standard deviation for the chemical and physical-chemical parameters over the six week period for the Organic (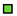), Conventional (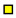) and Ground (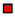) oils. Different letters indicate statistical differences (Tukey test’s *p* < 0.05).

In the case of the type of production system (Organic vs. Conventional), the effect was significant on the peroxide value (PV), the total phenols and the oxidative stability of oils (Table 1). The values averaged over the six week period for the Organic and Conventional oils are shown in Figure 1. Compared with the Conventional group, the Organic one had lower values for PV (9.63 ± 1.93 vs. 11.26 ± 2.12 mEq O_2_ kg^−1^), and higher for the total phenols (166.7 ± 15.0 vs. 149.0 ± 12.2 mg kg^−1^) and the oxidative stability (26.3 ± 2.1 vs. 22.9 ± 2.0 h). However, most quality parameters (free acidity and the extinction coefficients) were not affected (*p* > 0.730 for all of them) (Table 1). 

The lack of a marked effect on most of the quality parameters might be expected as the agronomic practices were very similar (no irrigation in both cases, and little vs. no use of phytosanitary chemicals) except for fertilisation. Currently, there is no general agreement on what the effect of the organic practices on the virgin olive oil quality is, apparently because of the difficulty of dealing with a relatively weak effect without excluding other environmental sources of variation. In this sense, a three-year study on the Leccino and Frantoio olive cultivars showed non-consistent differences in the oil quality parameters and phenol content due to the organic practices, suggesting that genotype and year-to-year variations in climate have a stronger effect [9]. However, both a decrease and an increase in oil quality have been reported in other studies. On the one hand, a decrease in oil quality (free acidity and *K*_270_) was reported and attributed to infestation and fungal infection in the organic fruits as a consequence of the absence of pesticides [21]. Likewise, lower phenol contents have been reported in organic Picual and Hojiblanca oils than in the conventional ones [13]. On the other hand, an increase in oil quality or/and in phenol content was also reported as a result of the organic practices. A study on Picual oil showed lower values for PV, greater stability and higher phenol content in the organic oil than in the conventional one [22], which is in line with our results for Verdial de Badajoz oil. More recent research on Leccio and Frantoio olive oils found no effect on the quality parameters but showed an increase in the phenol content, which was attributed to the decreased availability of soil nitrogen when using organic practices, as phenol content increases with decreased soil nitrogen availability [23], suggesting that, under controlled environmental conditions, the effect of the agronomic practices on plant metabolism is clear [8]. Similarly, organic practices on Kolovi olive orchards resulted in an increase in phenols, including luteolin, which was proposed as a marker for organic production [24]. Our results seem to confirm those results and show that the increase in phenols also happens in Verdial de Badajoz oil from unirrigated orchards. The increase in the total phenols (which have antioxidant activity) could explain the increase in oil stability and the decrease in PV. In any case, the moderate effect of the organic production on these parameters might indicate that no large differences might be expected in the volatile compounds.

#### 3.1.2. Effect of the Harvesting Method

The harvesting method affected all the parameters included in Table 1. Not only did the Ground oils (from ground-picked fruits) reach the highest and therefore worst values in all the quality parameters, but also the lowest in the total phenol content and oxidative stability (Figure 1). All the Ground oils, taken over the six-week period, exceeded considerably the 0.8% limit for free acidity for the extra virgin olive oil stated in European Commission Regulation (61/2011) [25], values being in the 2.4–5.3 range, and moreover all except the oil produced in week 2 exceeded the maximum allowance at least in one more quality parameter. Conversely, the two types of oils from tree-collected fruits (Conventional and Organic oils) were within the allowance limits for all the quality parameters. These results match those reported in Picual samples [26], with all the oils from ground-picked fruits exceeding the limit for free acidity and all the ones from tree-picked olives being below it. 

With respect to the total phenol content, ground-picked olives, which are more exposed to microbiological infection than tree-picked olives, might have yielded lower phenol concentrations due to the phenol decline that the microbiological activity causes, in particular in oleuropein derivatives [27]. Furthermore, since phenols protect oil from oxidation [28], a decrease in them would facilitate a decline in the oxidative stability of oil. These results show a considerable repercussion of the harvesting method on the Verdial de Badajoz oil quality over, regardless of the harvesting time, and confirm the fact that fruits collected from the ground result in poor quality olive oils [10,26]. Thus, clear differences in the volatile compound profile between the Ground oils and the others might be expected.

#### 3.1.3. Effect of the Harvesting Time

With respect to the harvesting time, the ANOVA revealed a slight effect, PV being the only parameter significant affected (Table 1). PV showed significant fluctuations throughout the six-week period instead of a steady trend (data not shown). Previous studies have reported that increased ripeness causes an undesirable increase in the values of the quality parameters, although with significant fluctuations, and a decrease in the oxidative stability and phenol content [12,29]. However, in our study harvesting was adjusted to real circumstances, where orchards management is set to harvest first the orchards which maturate first, as it is usually done in the commercial mills to achieve the best overall results, which could explain the lack of a clear trend. Our results show that in real conditions the harvesting time itself is not a critical quality factor for the Verdial de Badajoz oil as long as harvesting management is adequately set. The slight effect of harvesting time may anticipate slight changes in the volatile compound profile.

### 3.2. Volatile Compounds

A total of 145 volatile compounds were identified or tentatively identified in the headspace of the Verdial de Badajoz olive oils: 26 aldehydes, 13 ketones, 30 alcohols, 12 acids, 24 esters, 19 acyclic hydrocarbons, 13 cyclic hydrocarbons, four ethers and four other compounds (Table 2). The results from the three-way ANOVA show that few compounds were significantly affected by the organic production (34 out of 145), most of them (105 out of 145) were affected by the harvesting method and over half of them (83 out of 145) by the harvesting time (Table 2). Differences appeared not only in the lipoxygenase (LOX)-derived compounds (Table 3) but also in the fermentation (Table 4) and oxidation (Table 5) ones, as well as in all the chemical families of compounds (Table 2).

Regarding the type of production (Organic vs. Conventional), the modest effect found (Table 2) might be expected since the oils were just moderately different in the chemical and physical-chemical parameters (Table 1). Previous work on the effect of organic practices on the volatile compounds of olive oil has reported no consistent differences in a three-year study [9], but also a general rise [13,21], which could indicate that there might be further agronomic factors influencing the results.

Conversely, in the case of the harvesting method, the noticeable effect on the volatile compounds might be expected since the oils were markedly different in the chemical and physical-chemical parameters (Table 1). For most compounds, the largest abundances appeared in the Ground oils (Table 3, Table 4 and Table 5), which could be due to the mechanical damage caused by the drop of the fruits. The damage accelerates the decay process and makes easier the access of microorganisms to the fruits, whose infection results in changes in the volatile compound profile [27].

With regard to the harvesting time, the effect (Table 2) was stronger than expected taking into account the weak influence on the chemical and physical-chemical parameters (Table 1). In any case, a weak effect of the harvesting time was reported for Picual and Hojiblanca oils from irrigated orchards [13]. Our results for oil from unirrigated orchards suggest that the volatile compounds might be more affected by the harvesting time than what the chemical analyses may reveal. 

#### 3.2.1. LOX-Derived Volatile Compounds

Table 3 shows the results for the most representative C5 and C6 LOX volatile compounds affected by the organic production, the harvesting method, and/or the harvesting time. Those compounds were among the most abundant ones in the Verdial de Badajoz olive oil headspace, as it was previously reported for the oil from this cultivar [30] and others [3,4,10]. Most of those compounds have low odour-thresholds [3] and, therefore, could take part in oil flavour. In fact, C5 and C6 LOX compounds seem to contribute to the positive traits of olive oil [3,10]. The most abundant LOX compounds were (E)-hex-2-enal and (Z)-hex-3-en-1-ol, followed by hexan-1-ol and hexanal. It should be noted that hexanal, besides the LOX pathway, can be generated through oxidation reactions on linoleic acid [3], being involved in the rancid note of food when it appears at high concentrations. 

According to the ANOVA results (Table 2), the organic production affected eight out of the 13 LOX compounds included in Table 3, the harvesting method 12 out of 13 (all except hexanal), and the harvesting time 12 out of 13 (all except hexan-1-ol). Some LOX compounds were not significantly affected by any factors, such as pentan-2-one and 3-pentanol (Table 2). 

##### Effect of the Organic Production (Organic vs. Conventional)

The effect of the organic production was significant on four out of the six C5 compounds included in Table 3, and on four out of the seven C6 ones, according to the ANOVA results (Table 2). The effect was stronger than expected taking into account the relatively slight influence on the quality parameters (Table 1). Values for the C5 LOX compounds were generally lower in the Organic oils than in the Conventional ones, although in any case the Tukey test revealed only slight differences, especially on week 1. Different trends were found for important C6 compounds: (Z)-hex-3-en-1-ol tended to be more abundant in the Organic oils than in the Conventional ones (differences were significant in week 1, 4 and 6), whereas (Z)-hex-3-enal (weeks 1, 5 and 6), hexanal (weeks 3, 4, 5, 6) and (E)- hex-2-enal (weeks 1, 3, 4, 5, 6) showed the opposite trend. 

To date, the effect of the organic practices on the volatile compounds has been scarcely studied, and results are not completely consistent. In this sense, our results, from unirrigated Verdial de Badajoz orchards, show a similar trend for hexanal to results for oil from the Leccino and Frantoio cultivars also farmed in unirrigated orchards, although for the other compounds no clear trends were reported [9]. Conversely, higher abundances in hexanal in Organic than in Conventional oils from Picual and Hojiblanca olives from irrigated orchards have been also reported [13]. Therefore, our data might confirm that there is an effect of the organic production on some compounds, such as hexanal, but this effect might depend on other factors, such as irrigation or the olive cultivar.

##### Effect of the Harvesting Method

The significant effect of the harvesting method (Table 2) on all the C5 LOX compounds and six (all except hexanal) out of the seven C6 ones included in Table 3 matches the substantial effect found on the chemical and physical-chemical parameters (Table 1). The C5 LOX compounds were generally more abundant in the Conventional oils (from tree-picked fruits) than in the Ground ones. However, for the C6 LOX compounds a mixed trend was found. (Z)-hex-3-enal, (E)-hex-2-enal and (Z)-hex-3-en-1-ol, which have been related to the green attribute [10], were significantly more abundant in the Conventional group than in the Ground one. Conversely, hexan-1-ol and hexyl acetate tended to be more abundant over time in the Ground oils, and (E)-hex-2-en-1-ol did not show a steady trend. Hexan-1-ol is considered to elicit a no agreeable odour in oil [10] and, therefore, its increase might have a detrimental effect on oil quality. Hexyl acetate, which contributes to the fruity note, is an indicator of ripeness [3], and its precursor (E)-hex-2-en-1-ol [3] has been related to some defects [10,31]. The differences between the Conventional and Ground groups (Table 3) in the LOX compounds increased over time, the Tukey test revealing that it was on week 6 when the most C5 and C6 LOX compounds were influenced by the harvesting method (Table 3). (Z)-hex-3-enal and (E)-hex-2-enal were the compounds most affected, differences being significant in the Tukey test on all the weeks of sampling (Table 3). It should be noted that hexanal was not affected by the harvesting method. This result did not match a previous study reporting an increase in it in oil from ground-picked fruits [26]. However, hexanal content depends on the LOX pathway but also on oxidation reactions, and thus the lack of effect in our study (Table 2) may be explained by a counteracting effect of both pathways.

##### Effect of the Harvesting Time

According to the ANOVA results (Table 2), the effect of the harvesting time was significant on five out of the six C5 LOX compounds and six out of the seven C6 ones included in Table 3. It affected all the oil groups to a similar extent (Table 3). The effect was stronger on these compounds than on the chemical and physical-chemical parameters (Table 1). Most C5 LOX compounds fluctuated over time, without a consistent trend, although pentan-3-one and pent-1-en-3-ol decreased significantly as the season went on (Table 3). A similar pattern was reported for C5 LOX compounds in Arbequina and Chéttoui olive oils [4]. A general decrease was also found for the C6 compounds over time, especially for (Z)-hex-3-enal, (E)-hex-2-enal and (E)-hex-2-en-1-ol (Table 3), which are related to positive flavour traits [10]. These results for Verdial de Badajoz olive oil match previous results on other cultivars [4,29], although it has been pointed out that the decrease in C6 LOX compounds might not affect all cultivars [3]. The decrease over time was more marked in the Ground oils, which might indicate that harvesting late would add to the detrimental effect of harvesting from the ground.

#### 3.2.2. Fermentation Compounds

According to the ANOVA results (Table 2), the most important compounds related to the microbial activity were hardly affected by the organic practices (only ethanol was affected), but they were greatly influenced by the harvesting method (all the compounds included in Table 4 except 2-methylprop-2-enal) and the harvesting time (13 out of 18). 

##### Effect of the Organic Production (Organic vs. Conventional).

Except for ethanol, neither the non-phenolic fermentation compounds (including short-chain acids and alcohols and branched C3 and C4 compounds) nor the volatile phenols were affected by the organic practices (Table 2). This result suggests that differences in the agronomic practices such as fertilisation do not affect to a considerable extent the degradation reactions in which microorganisms can be involved once the fruits are released from the trees, which is in line with the moderate effect on the chemical and physical-chemical parameters (Table 1). Scarce information about the effect of the organic practices on the fermentation compounds is available, since most attention has been devoted to the LOX compounds [9], and no information is available about oils from unirrigated trees. For oil from irrigated orchards, a slight effect on the fermentation compounds was also reported for Hojiblanca (only 3-methylbut-2-en-1-ol was affected, without a consistent trend over time) and Picual oil (only methanol, 2-methylbutanal and 3-methylbut-2-en-1-ol were affected, with larger abundances in the organic oil) [13]. A larger content in 2-methylpropan-1-ol was reported in a group of organic oils than in the conventional ones, but also no differences were found in other fermentation compounds [21]. Our results show that the organic practices have not a noticeable effect on the fermentation compounds of Verdial de Badajoz olive oil from unirrigated orchards, which is partly in line with previous studies on other cultivars and irrigated orchards.

##### Effect of the Harvesting Method

Regarding the harvesting method, both the non-phenolic and phenolic fermentation compounds were markedly affected (12 out of 13, and all the phenols, respectively), the compounds being generally more abundant in the Ground oils than in the Conventional ones (Table 4). 

Almost all the non-phenolic compounds included in Table 4 (all except 2-methylprop-2-enal) were greatly affected by the harvesting method. Most of them were more abundant in the Ground oils than in the Conventional ones, although acetic acid followed the opposite trend. The generally higher values might be caused by the increased mechanical damage in the ground fruits and the subsequent opportunity for microbiological contamination and fermentation to occur. These results are in line with previous work reporting that fermentation compounds such as 2-methylbutan-1-ol and butan-1-ol were more abundant in oil from ground-picked olives than from tree-picked fruits [26]. Most of these compounds have low odour-thresholds [3], and 3-methylbutan-1-ol and short-chain acids have been related to the winey-vinegary and fusty defects [31].

With regard to the volatile phenols included in Table 4, they were all affected by the harvesting method (Table 2), all phenols being more abundant in the Ground oils. For three compounds (2-phenylethanol, 2-ethylphenol, and 4-ethyl-2-methoxyphenol) the differences were significant in the six weekly samplings (Table 4). Our results for Verdial de Badajoz oil are in line with the increase in the volatile phenols reported in Picual oils [26]. The volatile phenols are markers of fruit degradation [32] and, in fact, it has been suggested that 4-ethylphenol is a microbial metabolite from hydroxycinnamic acids [26]. They are abundant in oils with strong fusty, musty and muddy defects [31,33]. In addition to their relatively low odour-thresholds, phenols affect the release of some volatile compounds during consumption [34].

##### Effect of the Harvesting Time

The harvesting time had a significant effect (Table 2) on most non-phenolic (11 out of 13) and some phenolic (two out of five) fermentation compounds included in Table 4. Most of the non-phenolic compounds (all except 2-methylbutanal and 2-methylpropanoic acid) were affected by the harvesting time (Table 2). These compounds tended to fluctuate from week to week, although a general increase in ethanol, 2-methylpropanal and 3-methylbutanal was found. For most compounds the highest values tended to appear in weeks 3 and 4. The increase in ethanol over the harvesting season matches a rise in this compound found throughout fruit ripening [13]. This compound, which arises from fruit sugar fermentation and has been proposed as a marker of oil deterioration [13], has been related to the winey-vinegary defect [31]. Likewise, the branched aldehydes increased over time (Table 4). Although no increase was found in Hojiblanca and Picual oils [13], an increase in these undesirable compounds during fruit storage has been reported [4]. These compounds generally possess low odour-thresholds [3] and, in fact, they and their corresponding alcohols and acids are related to the fusty defect [10].

With regard to the volatile phenols included in Table 4, only 2-phenylethanol and 2-ethylphenol were affected according to the the ANOVA results (Table 2), with significant fluctuations over the six weeks consisting of an increase in week 3 and a subsequent decrease (Table 4). To our knowledge no information about the effect of either the harvesting time or fruit ripening on the volatile phenols of olive oil is available. As mentioned above, volatile phenols are related to oil degradation [4]. It was suggested that its formation may depend on the resistance of olives to microbial decay, and they have been related to the free acidity values [35]. In our study there was not a clear change in the quality parameters over time (Table 1) and the only parameter affected (PV) also showed fluctuations instead of a steady increase. Therefore, results for the volatile phenols (Table 4), which fit those for the quality parameters, could confirm that there was not a clear quality loss as the harvesting season elapsed.

#### 3.2.3. Oxidation-Derived Volatile Compounds

According to the ANOVA results (Table 2), most oxidation compounds included in Table 5 were significantly affected by the organic production (five out of the seven), all of them by the harvesting method, and two out of seven by the harvesting time. Some important oxidation compounds, such as (E)-dec-2-enal and the deca-2,4-dienal isomers, were not affected by any of the researched factors. 

##### Effect of the Organic Production (Organic vs. Conventional)

Regarding the organic production, the differences found in all the compounds except for heptanal and octanal were larger than expected considering the relatively modest effect found on the chemical and physical-chemical parameters (Table 1). (E)-hept-2-enal and nona-2,4-dienal, which possess low odour-thresholds [3], tended to be more abundant in the Conventional than in Organic oils over time. Nonetheless, there was not a steady trend in the other compounds, without significant differences between the Organic and Conventional oils most of the weeks (Table 5), which suggests that other environmental factors might have modulated the effect of the organic practices on them. Previous studies on organic practices have not paid attention to its effect on the volatile oxidation compounds, apart from hexanal, which is also a well-known LOX compound. In any case, (E)-hept-2-enal and nona-2,4-dienal possess low odour thresholds [3].

##### Effect of the Harvesting Method

The effect of the harvesting method was significant on all the oxidation compounds included in Table 5, according to the ANOVA results (Table 2). Heptanal, (E)-hept-2-enal, (E)-oct-2-enal, octanal and nonanal were generally more abundant in the oils from ground-picked fruits than in the ones from tree-picked fruits for all the sampling weeks (Table 4). Conversely, hexa-2,4-dienal and nona-2,4-dienal were less abundant in the Ground oils. 

Most of those compounds possess low odour-threshold [3]. (E)-hept-2-enal and (E)-2-octenal are among the main contributors to the rancid flavour in oil, and octanal and nonanal are also involved in this sensory defect [31]. In fact, octanal, nonanal and (E)-hept-2-enal are indicators of oxidative degradation [4]. Although oxidation compounds typically arise from oxidation reactions during oil storage, they are also formed as a consequence of fruit microbial activity [10,27], which is favoured when the fruits are collected from the ground. In fact, a higher content in octanal in oils from ground-picked fruits than from tree-picked ones was reported [26], although no information is available about the other compounds. Our results for the oxidation volatile compounds are in line with the marked effect found in the chemical and physical-chemical parameters (Table 1), and confirm for Verdial de Badajoz oil from unirrigated orchards the general rise in oxidation compounds when fruits are ground-collected.

##### Effect of the Harvesting Time

Regarding the harvesting time, only two out of the seven oxidation compounds included in Table 5 were affected, according to the ANOVA results (Table 2). There were significant fluctuations and also a slight decrease in hexa-2,4-dienal and (E)-hept-2-enal over the harvesting time regardless of the oil type (Table 5). However, most oxidation markers were not affected, harvesting over a six-week period having a slight influence on the oxidative volatile compounds of the Verdial de Badajoz oils, which could indicate that the harvesting time was suitably scheduled according to orchards ripening. Previous studies on the effect of harvesting during different periods did not included oxidation volatile compounds [13]. The slight effect is consistent with the results for the chemical and physical-chemical parameters, which were hardly affected (Table 1). Therefore, for unirrigated orchards, when timing is adequately set, only slight differences in the oxidation compounds are expected, the organic production and harvesting method having a much more noticeable effect.

### 3.3. Principal Component Analysis (PCA)

A Principal Component Analysis (PCA) was performed on the variables included in Table 1, Table 3, Table 4 and Table 5 to explore the relationships among them and the general effect of the organic practices, harvesting method and harvesting time. 

**Figure 2:**
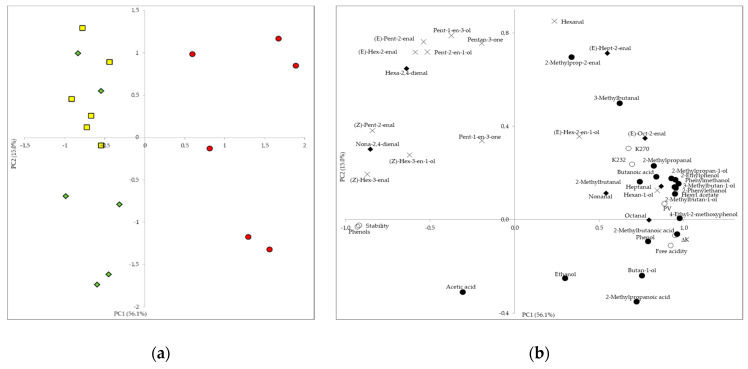
Projection of the oil samples (**a**) and variables from Table 1, Table 3, Table 4 and Table 5 (**b**) onto the space defined by the first two principal components (PC1/PC2). 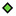: Organic; 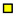 Conventional; 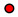 Ground. ο: Chemical and physical-chemical parameters; ×: LOX-derived volatile compounds; •: Fermentation compounds; ♦ Oxidation compounds.

Results show a different distribution of the samples according to the organic practices and harvesting method. The Ground oils were plotted in the positive PC1 semiaxis (Figure 2a), where the quality parameters and most of the fermentation and oxidation compounds had large loadings (Figure 2b). Conversely, the oils from fruits collected from the trees (both the Conventional and Organic oils) appeared in the negative PC1 semiaxis, where the total phenols, oil stability and most LOX compounds reached large absolute loadings (Figure 2b). Figure 2b shows that most LOX compounds were positively related to the total phenol content and oil stability (most of them had negative loadings, generally under −0.5) and negatively related to the quality parameters and most of the fermentation and oxidation compounds (most of them had positive loadings, generally above 0.5). Therefore, factors which favour fermentation or oxidation (for example, collecting the fruits from the ground) seem to hinder the LOX-pathway compounds.

In addition, the Organic oils tended to reach negative loadings in the PC2, whereas the Conventional ones tended to be displayed in the positive PC2 semiaxis. The variables with the largest absolute loadings in the PC2 were mostly LOX compounds, two oxidation compounds ((E)-hepten-2-al and hexa-2,4-dienal) and two fermentation compounds (2-methylprop-2-enal and 3-methylbutanal), all of them with positive values (Figure 2b). Therefore, these compounds seem to be more related to the Conventional than in the Organic oil according to the variability explained by the PC model.

Regarding the harvesting time, samples from the first weeks generally reached lower scores in the PC1 axis and higher in the PC2 axis, whereas the ones from the last weeks tended to follow the opposite trend. Therefore, at the beginning of the harvesting period, the oils tended to have higher scores in the variables positively related to oil quality, such as stability and phenol content, whereas at the end there was a slightly stronger relation to variables considered detrimental to oil quality (quality parameters, and fermentation and oxidation compounds). 

## 4. Conclusions

Results show that the organic practices in unirrigated orchards had a noticeable yet commercially modest effect on the chemical and physical-chemical parameters and the volatile compound profile. The effect was much less strong than that of the harvesting method, which affected severely the chemical and physical-chemical parameters, including the quality parameters (which are used in the official oil grading), and the volatile compounds. Conversely, the harvesting time in real conditions was revealed to be a factor with little repercussion on the oil quality parameters, which might be due to a suitable harvesting time schedule, although it had a noticeable effect on some important volatile compounds. Previous studies have not shown a consistent effect of the organic production on the olive oil characteristics, partly because of the difficulty of controlling other sources of variation. Our results, for oil obtained in a commercial mill, reveal an increase in the total phenols in the organic oil, which was previously reported at laboratory scale and attributed to the decreased availability of nitrogen due to the lack of chemical fertilisation. This result, obtained under controlled conditions (e.g., the same mill, the same area, the same harvesting time and method) could explain the increase in oil stability and the changes in the volatile compound profile. Considering that some studies involving irrigated orchards have not shown an increase in phenols, it would be advisable to perform further studies under controlled conditions to shed light on how irrigation may affect the effect of the organic practices, and on whether or not the organic fertilisation currently used in commercially exploited orchards causes a consistent decrease in soil nitrogen availability. That information would help understand the real defect of the organic production and how to deal with it.

## Figures and Tables

**Table 1:** *p*-Values from a three-way ANOVA (type of production, harvesting method and harvesting time) performed on the chemical and physical-chemical data of Verdial de Badajoz olive oil.

	Type of Production	Harvesting Method	Harvesting Time
Free acidity (%)	0.953	<0.001	0.388
PV (mEq O_2_ kg^−1^)	0.026	<0.001	0.006
*K*_232_	0.965	0.016	0.488
*K*_270_	0.823	0.024	0.506
Δ*K*	0.738	<0.001	0.276
Total phenols (mg kg−1)	0.039	<0.001	0.650
Oxidative stability (h)	0.006	<0.001	0.146

**Table 2:** Significance levels from a three-way ANOVA performed on the volatile compounds data * from the oil extracted from Verdial de Badajoz fruits farmed organically or conventionally (Prod.) and collected from the threes or from the ground (Meth.) over a six-week period (Week).

	*p*				*p*		
	Prod.	Meth.	Week		Prod.	Meth.	Week
*Aldehydes*				*Alcohols*			
Acetaldehyde ^c^	0.044	<0.001	0.138	**Ethanol ^c^**	<0.001	<0.001	<0.001
**2-Methylpropanal ^a^**	0.404	<0.001	<0.001	Propan-1-ol ^a^	0.497	<0.001	0.013
**2-Methylprop-2-enal ^b^**	0.096	0.067	0.006	**2-methylpropan-1-ol ^b^**	0.534	<0.001	0.001
Butanal ^a^	0.181	0.644	0.709	**Butan-1-ol ^a^**	0.667	<0.001	0.011
But-2-enal ^a^	0.400	0.071	<0.001	**Pent-1-en-3-ol ^a^**	0.008	0.001	<0.001
**3-Methylbutanal ^a^**	0.549	<0.001	<0.001	Pentan-3-ol ^b^	0.279	0.750	0.443
**2-Methylbutanal ^a^**	0.472	0.003	0.191	3-Methylbut-3-en-1-ol ^b^	0.311	<0.001	<0.001
**(Z)-Pent-2-enal ^b^**	0.006	<0.001	0.046	**3-Methylbutan-1-ol ^a^**	0.332	<0.001	<0.001
**(E)-Pent-2-enal ^a^**	0.004	<0.001	0.001	**2-Methylbutan-1-ol ^a^**	0.287	<0.001	<0.001
**(Z)-Hex-3-enal ^b^**	0.003	<0.001	<0.001	Pentan-1-ol ^a^	0.779	0.002	0.203
**Hexanal ^a^**	<0.001	0.797	<0.001	**(Z)-Pent-2-en-1-ol ^b^**	0.082	0.001	<0.001
**(E)-Hex-2-enal ^a^**	<0.001	<0.001	0.001	**(Z)-Hex-3-en-1-ol ^a^**	0.009	<0.001	0.001
**Heptanal ^a^**	0.556	<0.001	0.058	**(E)-Hex-2-en-1-ol ^b^**	0.237	0.004	0.003
Hexa-2,4-dienal ^a^	0.025	<0.001	0.001	**Hexan-1-ol ^a^**	0.642	<0.001	0.092
(E)-Hept-2-enal ^a^	0.001	0.002	0.001	Heptan-2-ol ^a^	0.685	0.096	0.284
Benzaldehyde ^a^	0.688	<0.001	0.006	Phenol ^b^	0.336	<0.001	0.065
**Octanal ^a^**	0.449	<0.001	0.709	Heptan-1-ol ^a^	0.974	<0.001	0.090
**(2E)-Oct-2-enal ^a^**	0.011	0.001	0.826	Oct-1-en-3-ol ^a^	0.478	<0.001	0.063
(3E)-Non-3-enal ^b^	0.548	<0.001	0.723	Octan-3-ol ^b^	0.001	<0.001	<0.001
Nonanal ^a^	0.048	0.037	0.182	Octan-2-ol ^a^	0.017	<0.001	<0.001
(E)-Non-2-enal ^a^	0.357	0.001	0.011	2-Ethylhexan-1-ol ^b^	0.681	0.081	0.044
**Nona-2,4-dienal ^b^**	<0.001	<0.001	0.266	Phenylmethanol ^b^	0.933	<0.001	0.150
(E)-Dec-2-enal ^b^	0.249	0.226	0.370	(Z)-Oct-2-en-1-ol ^b^	0.971	0.004	0.570
(E,Z)-Deca-2,4-dienal ^b^	0.721	0.442	0.245	Octan-1-ol ^a^	0.555	<0.001	0.542
(E,E)-Deca-2,4-dienal ^b^	0.790	0.567	0.491	Phenylethanol ^b^	0.959	<0.001	0.008
Undecenal ^b^	0.274	0.657	0.549	(Z)-Non-3-en-1-ol ^b^	0.694	<0.001	<0.001
*Ketones*				2-Ethylphenol ^b^	0.928	<0.001	0.047
Propan-2-one ^c^	0.004	0.490	0.038	Nonan-1-ol ^a^	0.502	<0.001	0.246
Butan-2-one ^a^	0.755	<0.001	0.158	Decan-1-ol ^b^	0.470	0.012	0.165
Pent-1-en-3-one ^a^	0.031	0.045	0.066	4-Ethyl-2-methoxyphenol ^b^	0.767	<0.001	0.100
**Pentan-2-one ^a^**	0.675	0.196	0.320	*Acids*			
**Pentan-3-one ^b^**	0.102	0.022	<0.001	**Acetic acid ^a^**	0.576	0.020	<0.001
3-Hydroxybutan-2-one ^b^	0.410	0.001	<0.001	**2-Methylpropanoic acid ^a^**	0.916	0.001	0.317
4-Methylpentan-2-one ^b^	0.556	0.026	0.005	**Butanoic acid ^a^**	0.630	<0.001	0.024
2-Methylpentan-3-one ^b^	0.971	<0.001	0.325	**2-Methylbutanoic acid ^a^**	0.671	<0.001	0.014
Hexan-2-one ^a^	0.851	0.002	0.338	Hexanoic acid ^a^	0.570	<0.001	0.094
Heptan-2-one ^a^	0.731	<0.001	0.236	Heptanoic acid ^a^	0.482	0.318	0.511
Octan-3-one ^a^	0.595	<0.001	0.002	Octanoic acid ^a^	0.376	0.259	0.040
Octan-2-one ^b^	0.728	<0.001	0.213	Nonanoic acid ^a^	0.779	0.215	0.607
Nonan-2-one ^b^	0.550	<0.001	0.044	Decanoic acid ^a^	0.272	0.388	0.525
*Esters*				Undecanoic acid ^b^	0.402	0.085	0.659
Methyl acetate ^b^	0.003	0.043	<0.001	Dodecanoic acid ^b^	0.681	0.391	0.632
Ethyl acetate ^b^	0.001	<0.001	<0.001	Tridecanoic acid ^b^	0.084	0.647	0.701
Methyl propanoate ^b^	0.086	<0.001	<0.001	*Acyclic hydrocarbons*			
Ethyl propanoate ^a^	0.508	<0.001	0.018	Pentane ^a^	0.015	<0.001	<0.001
Methyl butanoate ^a^	0.048	<0.001	0.001	2-Methylbut-2-ene ^b^	0.806	0.597	0.002
Ethyl 2-methylpropanoate ^a^	0.565	<0.001	<0.001	Penta-2,3-diene ^b^	0.015	<0.001	<0.001
Methyl 2-methylbutanoate ^a^	0.748	<0.001	0.017	Hexane ^a^	<0.001	0.603	0.026
Methyl pentanoate ^a^	0.055	0.016	<0.001	2-Methylpent-2-ene ^b^	0.724	<0.001	0.006
Ethyl 2-methylbutanoate ^a^	0.205	0.085	<0.001	2-methylpenta-1,3-diene ^b^	0.967	<0.001	0.003
3-Methylbutyl acetate ^b^	0.119	<0.001	0.020	2-Methylhexane ^b^	0.194	0.239	0.050
2-Methylbutyl acetate ^b^	0.040	<0.001	0.011	5-methylhex-1-ene ^b^	0.021	0.981	0.685
2-Methylpropyl 2-methylpropanoate ^b^	0.145	<0.001	0.006	3-Methylhexane ^b^	0.013	0.382	0.004
Methyl hexanoate ^a^	0.003	0.001	0.041	Hept-1-ene ^b^	0.522	<0.001	0.074
Methyl Hex-4-enoate ^b^	0.867	0.639	0.455	Heptane ^a^	0.511	0.001	0.068
Ethyl hexanoate ^a^	0.001	<0.001	<0.001	Oct-1-ene ^b^	0.706	<0.001	0.006
(E)-Hex-3-enyl acetate ^b^	<0.001	0.026	0.023	Octane ^a^	0.335	<0.001	0.003
**Hexyl acetate ^b^**	0.799	<0.001	0.001	(Z)-Oct-2-ene ^b^	0.441	<0.001	0.004
3-Methylbutyl 2-methylpropanoate ^b^	0.348	<0.001	0.022	(E)-Oct-2-ene ^b^	0.477	<0.001	0.001
Methyl heptanoate ^b^	0.091	<0.001	0.554	(E)-β-Ocimene ^b^	0.118	0.549	0.160
Methyl benzoate ^b^	0.224	0.008	0.777	Alloocimene ^b^	0.112	0.497	0.001
Methyl octanoate ^b^	0.263	0.001	0.022	Dodecane ^a^	0.617	0.751	<0.001
Ethyl benzoate ^b^	0.301	<0.001	0.369	(E,E)-α-farnesene ^b^	0.029	0.513	<0.001
Ethyl octanoate ^b^	0.812	0.010	0.352	*Cyclic hydrocarbons*			
Methyl 2-methoxybenzoate ^b^	0.531	0.334	0.372	Toluene ^b^	0.934	<0.001	0.095
*Others*				1,4-Dimethylbenzene ^b^	0.129	<0.001	0.028
Diethyl ether ^a^	0.556	0.191	0.031	Styrene ^b^	0.147	<0.001	0.001
2-Ethoxy-2-methylpropane ^b^	0.819	0.001	0.039	1,3-Dimethylbenzene ^b^	0.892	0.001	0.032
1-Methoxyhexane ^b^	0.293	<0.001	0.071	1-Ethyl-3-methylbenzene ^b^	0.026	0.200	0.186
Methoxybenzene ^b^	0.015	0.228	0.126	1,2,4-Trimethylbenzene ^b^	0.581	0.095	0.071
Dimethyl sulfide ^b^	0.708	<0.001	0.009	1,3-Diethylbenzene ^b^	0.100	0.030	<0.001
4-Methyl-2,3-dihydrofuran ^b^	0.372	0.482	0.362	l-Limonene ^a^	0.691	<0.001	0.010
Γ-Hexalactone ^b^	0.001	<0.001	0.016	3-(4-Methylpent-3-enyl)furan ^b^	0.290	<0.001	0.011
δ-Octalactone ^b^	0.153	0.202	0.012	α-Copaene ^b^	0.976	0.359	0.354
				γ-Cadinene ^b^	0.907	0.561	0.283
				Eremophilene ^b^	0.251	<0.001	0.224
				α-Muurolene ^b^	0.843	0.037	0.391

* The compound was identified by comparing it with: ^a^ the MS and LRI of the reference compound; ^b^ MS and LRI from literature; ^c^ the MS and retention time of the reference compound. Bold: compounds included on Table 3, Table 4 and Table 5.

**Table 3:** Results (means and significance *) for the most representative C5 and C6 LOX volatile compounds of the Verdial de Badajoz oil headspace significantly affected by the organic production, harvesting method and/or harvesting time according to Table 2.

		Week 1	Week 2	Week 3	Week 4	Week 5	Week 6
(Z)-Pent-2-enal	Organic	0.42	0.45a	0.21	0.50a	0.20b	0.26ab
	Conventional	AB0.55	C0.3a	BC0.39	AB0.57a	A0.61a	ABC0.48a
	Ground	A0.31	AB0.06b	AB0.07	AB0.06b	B0.04c	AB0.05b
(E)-Pent-2-enal	Organic	A2.86ab	A2.46a	B0.77b	A2.48ab	B0.92b	B1.39b
	Conventional	AB2.97a	C1.65ab	BC2.24a	A3.20a	ABC2.51a	ABC2.42a
	Ground	A2.04b	ABC1.19b	A2.03a	AB1.61b	C0.42c	BC0.89c
Pent-1-en-3-one	Organic	A3.69	A4.4	B0.78	A4.82	B1.61	A4.04
	Conventional	5.50	1.26	5.44	7.33	5.15	7.46
	Ground	1.77	2.18	0.86	5.86	5.42	4.24
Pentan-3-one	Organic	A21.37	C7.92b	BC11.40b	AB17.42	C6.64b	C6.40ab
	Conventional	B13.65	B14.48a	A19.85a	B15.94	C9.78a	C9.34a
	Ground	AB13.38	AB11.57a	A15.80ab	AB12.68	BC7.49ab	C4.99b
Pent-1-en-3-ol	Organic	A5.62	B1.91	B2.06c	AB3.16ab	B1.14c	B1.49b
	Conventional	AB3.65	B3.12	A4.30a	A4.58a	B2.63a	B2.78a
	Ground	A3.37	BC2.35	AB3.21b	CD2.14b	DE1.43b	E0.81c
Pent-2-en-1-ol	Organic	A7.49	AB3.48	B2.97	AB3.95ab	B1.82	B2.65ab
	Conventional	4.76	4.11	4.94	5.40a	3.61	4.18a
	Ground	4.67	3.52	2.85	3.14b	1.67	1.62b
(Z)-Hex-3-enal	Organic	A10.42ab	A11.29a	BC5.63a	B7.29a	C5.14b	BC6.75b
	Conventional	A16.88a	B8.08a	B5.31a	B9.65a	AB11.07a	AB11.28a
	Ground	A3.51b	B1.06b	B0.72b	B0.74b	B0.07b	B0.20c
Hexanal	Organic	AB28.56	BC20.80b	C11.60b	A35.69b	BC18.16b	AB26.3b
	Conventional	BC31.70	C28.62ab	BC32.26a	A43.36a	ABC36.47a	AB38.49a
	Ground	AB41.49	ABC33.82a	ABC35.81a	A43.29a	C24.69b	BC28.51b
(E)-Hex-2-enal	Organic	A63.33b	A61.55a	B23.52c	A56.35b	B24.73b	B33.97b
	Conventional	AB82.37a	B68.07a	AB85.02a	A100.50a	AB83.06a	AB83.54a
	Ground	A74.71ab	B41.51b	B38.57b	B42.47c	C6.32c	C13.78c
(Z)-Hex-3-en-1-ol	Organic	AB73.07a	D44.12	BC67.15a	A85.86a	CD55.31a	BC63.62a
	Conventional	54.92b	56.64	64.12a	59.07b	47.84a	49.89b
	Ground	A55.38b	AB46.14	AB44.90b	BC42.64c	CD31.74b	D27.31c
(E)-Hex-2-en-1-ol	Organic	A22.64b	C4.86c	B12.95b	B12.53	C4.23c	C4.09c
	Conventional	B10.04a	A17.57b	A23.03a	B10.69	B8.78b	B9.49a
	Ground	B27.97b	A36.20a	C21.11a	D12.87	C20.85a	D7.37b
1-Hexanol	Organic	35.13a	17.65b	29.85b	35.39ab	24.9b	28.71b
	Conventional	24.03b	33.22a	35.56b	26.40b	22.30b	23.05b
	Ground	35.13a	39.87a	52.68a	41.82a	52.49a	36.18a
Hexyl acetate	Organic	0.33	0.31	0.40b	0.56b	0.35b	0.51b
	Conventional	B0.26	AB0.49	A0.72b	AB0.44b	B0.30b	AB0.34b
	Ground	C0.72	BC0.81	A1.58a	AB1.35a	ABC1.06a	ABC1.20a

* Different letters in the same row (A–C) or the same column (a–c) indicate statistical differences (Tukey test’s *p* < 0.05). Results are expressed as AU × 10^−6^.

**Table 4:** Results (means and significance *) for the most representative fermentation compounds of the Verdial de Badajoz oil headspace significantly affected by the organic production, harvesting method and/or harvesting time according to Table 2.

		Week 1	Week 2	Week 3	Week 4	Week 5	Week 6
2-Methylpropanal	Organic	CD0.56	D0.31	CD0.66b	A1.72	BC1.03	AB1.45a
	Conventional	B0.46	B0.42	AB1.09b	A1.35	AB0.74	AB0.64b
	Ground	B1.35	B1.09	A3.65a	AB2.63	AB1.70	AB1.90a
2-Methylprop-2-enal	Organic	AB 0.38b	AB 0.35	B0.07c	AB 0.32b	AB0.18b	A0.48
	Conventional	AB0.46b	B0.26	AB0.40b	A0.66b	AB0.47a	AB0.45
	Ground	AB1.06a	C0.46	BC0.59a	A1.17a	C0.05b	C0.40
3-Methylbutanal	Organic	C0.99b	C0.65b	C0.79b	A4.67b	B2.43b	B2.99a
	Conventional	BC1.43b	C1.27b	A3.90a	A3.52c	B1.96c	BC1.88b
	Ground	BC4.13a	CD2.60a	AB5.40a	A6.37a	CD3.30a	D1.89b
2-Methylbutanal	Organic	2.68	0.91	2.69	4.81	3.79	6.41
	Conventional	2.62	2.79	3.95	2.19	3.06	3.43
	Ground	4.45	3.83	7.05	8.13	5.65	3.56
Ethanol	Organic	D59.14a	E43.79a	C91.84a	B112.40a	AB121.59a	A126.34b
	Conventional	D15.30	D21.38b	C52.41b	A89.89b	A80.90b	B69.13c
	Ground	C60.28a	C49.84a	A120.13a	B84.87b	C55.72c	A139.93a
2-Methylpropan-1-ol	Organic	B1.06b	B0.53b	A2.64b	A2.81b	A2.05b	A2.25b
	Conventional	C0.80b	C1.02b	A2.71b	B1.90c	C1.01c	C1.05c
	Ground	C6.05a	C6.39a	A16.34a	AB11.92a	BC8.54a	C5.69a
Butan-1-ol	Organic	0.43	0.73	0.35	0.49	1.17	0.96
	Conventional	0.35	0.22	0.76	0.74	0.87	0.70
	Ground	0.60	0.98	1.71	1.26	2.45	1.14
3-Methylbutan-1-ol	Organic	C3.36b	C1.96b	AB9.87b	A11.85b	B8.47b	AB10.13b
	Conventional	C3.00b	C4.14b	A10.42b	B7.09b	C4.14c	C4.45c
	Ground	B19.83a	B20.58a	A50.92a	A40.31a	B25.83a	B26.33a
2-Methylbutan-1-ol	Organic	D1.54b	D0.79b	C4.01b	A6.04b	BC4.60b	AB5.29b
	Conventional	C1.12b	C1.89b	A5.80b	B3.18c	C1.78c	C1.93c
	Ground	C8.53a	C9.58a	A23.3a	AB17.81a	BC14.48a	C10.87a
Acetic acid	Organic	C3.25	B17.76a	A64.99a	AB9.35	AB6.6a	AB9.45a
	Conventional	B24.18	B19.54a	A38.92b	C4.26	C3.9b	C5.94b
	Ground	2.04	3.98b	8.43c	5.77	4.21b	8.43ab
2-Methylpropanoic acid	Organic	0.16	0.11b	0.22b	0.24b	0.10	0.24b
	Conventional	0.12	0.19b	0.31b	0.05b	0.00	0.12c
	Ground	0.51	0.62a	1.16a	0.80a	4.12	2.74a
Butanoic acid	Organic	0.29	0.04b	0.28b	0.39b	0.09b	0.94
	Conventional	B0.00	B0.19b	A0.77b	B0.23b	B0.05b	B0.18
	Ground	B0.44	B1.31a	A2.89a	AB1.66a	B1.28a	B0.66
2-Methylbutanoic acid	Organic	B0.00b	B0.01b	A0.29b	B0.00	AB0.12b	AB0.11b
	Conventional	0.08b	0.00b	0.00b	0.11	0.07b	0.13b
	Ground	B0.37a	AB0.59a	A1.01a	AB0.76	AB0.78a	AB0.66a
Phenylmethanol	Organic	1.10b	1.14	1.09	1.89	0.63b	2.17b
	Conventional	1.32b	1.26	2.35	1.14	0.82b	0.85b
	Ground	3.81a	4.24	8.06	6.97	5.31a	5.29a
2-Phenylethanol	Organic	0.44b	0.35b	0.66b	0.9b	0.66b	1.03b
	Conventional	B0.18b	B0.64b	A1.35b	AB0.8b	B0.38b	B0.57b
	Ground	AB6.58a	AB7.37a	A9.54a	AB8.25a	B5.08a	AB7.55a
Phenol	Organic	0.15b	0.07b	0.06	0.02	0.00b	0.03c
	Conventional	0.16b	0.05b	0.09	0.02	0.07b	0.12b
	Ground	BC0.26a	BC0.32a	B0.34	C0.15	A0.52a	B0.33a
2-Ethylphenol	Organic	0.00b	0.41b	0.00b	1.87b	0.84b	0.03b
	Conventional	B0.31b	B0.04b	A2.06b	B0.58b	B0.45b	B0.28b
	Ground	6.52a	9.38a	18.42a	14.04a	8.1a	10.47a
4-Ethyl-2-methoxyphenol	Organic	0.00b	0.00b	0.05b	0.06b	0.04b	0.20b
	Conventional	0.00b	0.05b	0.00b	0.02b	0.09b	0.03b
	Ground	1.57a	2.23a	2.44a	2.38a	2.35a	2.26a

* Different letters in the same row (A–C) or the same column (a–c) indicate statistical differences (Tukey test’s *p* < 0.05). Results are expressed as AU × 10^−6^.

**Table 5:** Results (means and significance *) for the most representative oxidation compounds of the Verdial de Badajoz oil headspace significantly affected by the organic production, harvesting method and/or harvesting time according to Table 2.

		Week 1	Week 2	Week 3	Week 4	Week 5	Week 6
Heptanal	Organic	1.90b	1.41	1.62	2.45ab	2.36	2.69
	Conventional	1.81b	2.34	1.77	2.23b	2.31	2.81
	Ground	3.66a	2.48	3.80	4.38a	4.14	3.05
Hexa-2,4-dienal	Organic	A4.95a	BC3.68a	D1.52b	AB4.28a	D1.20b	C2.83a
	Conventional	B3.44ab	B3.33a	A5.20a	A5.34a	B3.13a	B3.04a
	Ground	AB2.02b	ABC1.16b	A2.33b	A2.11b	C0.60b	BC0.81b
(E)-Hept-2-enal	Organic	AB6.64b	AB6.54b	D2.60c	A7.96b	C4.40b	BC5.91b
	Conventional	A9.16ab	B6.62b	AB7.96b	A8.87b	AB7.69a	AB8.56a
	Ground	B11.69a	C9.62a	B12.54a	A14.92a	D4.97b	C9.43a
Octanal	Organic	2.52b	1.66	2.09c	3.09	2.51b	3.91
	Conventional	2.02b	4.31	3.27b	2.77	2.29b	3.14
	Ground	4.94a	3.14	4.54a	4.49	6.43a	4.65
(E)-Oct-2-enal	Organic	0.79	0.65	0.40c	0.85b	0.69	0.69b
	Conventional	0.92	1.27	1.00b	0.76b	0.90	0.83b
	Ground	1.23	1.16	1.42a	1.53a	1.03	1.50a
Nonanal	Organic	8.75b	6.90	7.90	10.52	11.86	13.04
	Conventional	10.44b	15.90	9.14	12.88	10.73	16.36
	Ground	20.33a	12.45	12.61	15.34	16.57	15.60
Nona-2,4-dienal	Organic	0.84b	0.95	0.35b	1.06a	0.72b	0.91b
	Conventional	1.10a	1.11	1.32a	1.29a	1.15a	1.45a
	Ground	0.45c	0.27	0.11b	0.12b	0.13c	0.03c

* Different letters in the same row (A–C) or the same column (a–c) indicate statistical differences (Tukey test’s *p* < 0.05). Results are expressed as AU × 10^−6^.
